# The effects of pre- and post-exercise consumption of multi-ingredient performance supplements on cardiovascular health and body fat in trained men after six weeks of resistance training: a stratified, randomized, double-blind study

**DOI:** 10.1186/1743-7075-10-39

**Published:** 2013-05-16

**Authors:** Michael J Ormsbee, Dennison David Thomas, William Kyle Mandler, Emery G Ward, Amber W Kinsey, Lynn B Panton, Timothy P Scheett, Shirin Hooshmand, Emily Simonavice, Jeong-Su Kim

**Affiliations:** 1 Dept. of Nutrition, Food and Exercise Sciences, The Florida State University, 120 Convocation Way, 430 Sandels Building, Tallahassee, FL 32306, USA; 2 Dept. of Health and Human Performance, College of Charleston, 66 George St, Charleston, SC 29424, USA; 3 School of Exercise & Nutritional Sciences, San Diego State University, San Diego, CA 92182, USA; 4 Dept. of Kinesiology, Georgia College and State University, Milledgeville, GA 31061, USA

**Keywords:** Weight lifting, Supplementation, Regional body fat, Fat-free mass, Health

## Abstract

**Background:**

The cardiovascular (CV) and metabolic health benefits or risks associated with consumption of multi-ingredient performance supplements (MIPS) in conjunction with periodized resistance training (RT) in resistance-trained men are unknown. This population is a major target audience for performance supplements, and therefore, the purpose of this study was to investigate the combined effect of RT and commercially available pre- and post-exercise performance supplements on CV health and body fat in resistance-trained men.

**Methods:**

Twenty-four resistance-trained men completed six weeks (three times/week) of periodized RT while either ingesting SHOT 15-min pre-exercise and SYN immediately post-exercise (multi-ingredient performance supplement group: MIPS) or an isocaloric maltodextrin placebo 15-min pre-exercise and immediately post-exercise (Placebo group). Before and after six weeks of RT and supplementation, resting heart rate (HR), blood pressure (BP), total body fat, android fat, gynoid fat, fat-free mass (FFM) and fasting blood measures of glucose, lipids, nitrate/nitrite (NOx), cortisol and high sensitivity C-reactive protein (hs-CRP) were measured. Statistical analysis was conducted using a one-way ANOVA for baseline differences and a 2 × 2 (group × time) repeated measures ANOVA and Tukey post-hoc tests where appropriate. Significance was set at p < 0.05.

**Results:**

There was no group × time interaction for HR, BP, blood glucose, lipids, NOx, hs-CRP, cortisol concentrations or body fat. However, there was a time effect where significant decreases in body fat (mean ± SD; MIPS: -1.2 ± 1.2%; Placebo: -0.9 ± 1.1%), android fat (MIPS: -1.8 ± 2.1%; Placebo: -1.6 ± 2.0%), and gynoid fat (MIPS: -1.3 ± 1.6%; Placebo: -1.0 ± 1.4%) for both groups were observed. FFM increased in both groups, and a group × time interaction was observed with MIPS increasing significantly more than the Placebo group (4.2% vs. 1.9%).

**Conclusions:**

Six weeks of MIPS ingestion and periodized RT does not alter CV health parameters or blood indices of health or body fat more than a Placebo treatment in healthy, resistance-trained men. However, MIPS significantly increased FFM more than Placebo.

## Background

Nutritional supplements intended for consumption before and after resistance training (RT) to improve performance are extremely popular among young men and athletes [1,2]. Given that RT has been shown to increase muscle fiber size and strength in men and women [3] with a concomitant increase in lipolysis and fat oxidation [4], it is not surprising that performance supplements are consumed in conjunction with RT in an attempt to improve body composition and athletic performance. The components of these popular multi-ingredient performance supplements (MIPS; e.g. whey protein, casein protein, branched-chain amino acids (BCAA’s), creatine, β-alanine, caffeine, and L-arginine) have been studied individually for their ergogenic effects [5-8]; however, the health and safety outcomes of these supplements are not well known when consumed in combination in a well-trained population. Caffeine alone has been shown to increase resting blood pressure (BP) acutely [9], and potentially chronically [10]. Systolic blood pressure (SBP) and heart rate (HR) have also been shown to increase during RT with acute caffeine supplementation in resistance-trained men [11]. However, recent studies evaluating supplements that contain caffeine do not report measures of BP or HR [12-14].

Multiple side effects have been demonstrated with the use of MIPS. Shelmadine et al. [12] investigated eighteen untrained men following 28 days of RT (4×/week) when consuming NO-Shotgun® (Vital Pharmaceuticals, Davie, FL) containing a blend of protein, BCAA’s, creatine, β-alanine, caffeine, and L-arginine consumed 30 minutes before RT compared to an isocaloric placebo (maltodextrin) and found side effects including dizziness, nausea, headache, and shortness of breath in 44% (n = 4 of 9) of this group’s participants. However, 44% (n = 4 of 9) of participants taking the placebo also experienced side effects of nausea and shortness of breath. Interestingly, the authors did not report BP or HR data, which may be important given these side effects and media reports portraying these pre- and post-RT products as dangerous. However, fasting blood lipid profile and glucose were not altered in either group. Significant increases were reported in fat-free mass (FFM) with no significant changes in fat mass. This improvement in body composition likely overrides the potential negative consequences to using MIPS supplements for the young athletes targeted by such products [1,2] as anecdotal evidence suggests that young weight training men are most concerned with adding muscle mass.

In a follow-up study, Spillane et al. [13] investigated untrained men and provided NO-Shotgun® (Vital Pharmaceuticals, Davie, FL) pre-RT and added consumption NO-Synthesize®, (Vital Pharmaceuticals, Davie, FL) immediately post-RT and on all non-RT days. The authors reported similar side effects for these untrained participants including nausea, rapid HR, and shortness of breath regardless of being in the MIPS or placebo groups [13]. Again, BP and HR data were not reported but significant (p = 0.023) increases in FFM were found in the supplement group when compared to the placebo group after 28 days (mean ± SD; Supplement: Day 0, 57.8 ± 6.4 kg vs. Day 28, 59.9 ± 7.6 kg; placebo: Day 0, 56.4 ± 10.3 kg vs. Day 28, 57.0 ± 9.9 kg). In addition, whole blood and serum clinical chemistry markers, regardless of group, remained in the normal ranges in this study [13]. The health implications of MIPS use in trained men, however, deserve further investigation.

Therefore, the purpose of the present study was to evaluate the effects of six weeks of pre- and post-RT MIPS consumption with periodized RT on cardiovascular (CV) and metabolic health and body fat in resistance-trained men. We hypothesized that the inclusion of a pre- and post-RT MIPS with resistance-trained participants would not alter CV markers of health, but would improve cortisol, mood state, and body fat more than a placebo supplement.

## Methods

### Participants

Twenty-nine healthy, resistance trained (≥3× per week; training for ≥12 months) men (mean ± SD; age, 24.0 ± 2.3 years; height, 180.5 ± 5.8 cm; body mass, 83.7 ± 0.5 kg; body mass index, BMI, 25.5 ± 2.2 kg · m^2^) began this study. However, five (MIPS: n = 1; Placebo: n = 4) withdrew over the course of the six-week protocol (three for personal reasons, one was found to be a smoker, and one did not follow the RT protocol). Participants were non-smokers and had no known existing diseases, musculoskeletal disorders or injuries, uncontrolled hypertension (BP > 140/90 mmHg), uncontrolled cholesterol/blood lipid levels, use of cholesterol medication, or dairy allergies as assessed by a medical history questionnaire. Those reporting anabolic steroid use on the medical history form were excluded. Use of other performance supplements (e.g. caffeine, β-alanine, and creatine monohydrate) required a four-week washout period [15] prior to participation (excluding multivitamins). Participants were instructed not to take any supplement, other than the provided pre- and post-RT supplements, for the duration of the study. All procedures involving human subjects were approved by The Florida State University Human Subjects Institutional Review Board in accordance with the Helsinki Declaration. Written informed consent was obtained prior to participation.

### Study design and supplementation protocol

Participants were randomly assigned to one of two groups in this placebo-controlled, double blind, six-week protocol after being stratified by isometric maximal voluntary contraction strength (dominant quadriceps) to FFM ratio. Participants in the MIPS group consumed 21 g of NO-Shotgun® (SHOT) which contained 72 kcals, 18 g protein, 0 g carbohydrates, 0 g fat, caffeine ~190 mg; containing a proprietary blend of whey protein, BCAA’s, creatine, β-alanine, and L-arginine (Vital Pharmaceuticals, Davie, FL) 15 minutes before RT and 21 g of NO-Synthesize® (SYN) which contained ~80 kcals, 20 g protein, 0 g carbohydrate, 0 g fat; containing a proprietary blend of whey protein, BCAA’s, creatine, β-alanine, and L-arginine (Vital Pharmaceuticals, Davie, FL) immediately after RT. Participants in the Placebo group consumed 21 g of an isocaloric, flavor-matched maltodextrin placebo 15 minutes before RT and 21 g immediately post-RT. All supplements were distributed in identical single-serving Ziploc® bags before and after each workout and research personnel watched as each participant consumed the supplement on all training days. For all non-training days, participants were given single-servings of the supplement in Ziploc® bags to take home and consume. In the morning for non-training days, MIPS consumed 21 g/day of SYN and the Placebo group consumed 21 g/day of Placebo. These empty bags were collected and participants were asked to verbally verify supplement compliance on each workout day (our acceptable compliance margin was set to >80% to be included in the data analysis).

### Laboratory testing

Laboratory testing took place only before and after the six-week intervention. Profile of mood state [16], HR, BP, body composition, and blood measures took place following a 10-hour fast and a 24-hour restriction of physical activity, caffeine, and alcohol intake at baseline and after six weeks of RT and supplement intervention. Six-week testing (post) took place 48 hours after conclusion of RT to avoid any residual impact of the last training session on dependent variables including blood markers of inflammation (hs-CRP) and catabolism (cortisol) which may be elevated following an acute bout of RT, but not as a result of RT over the entire study period.

### Profile of mood states (POMS)

POMS [16] scores were measured upon arriving to the laboratory and were used to measure mood before and after the six-week intervention. These scores are compiled based on responses to 65 mood descriptors and divided into six categories describing tension, depression, anger, vigor, fatigue, and confusion. In addition, total mood score was calculated. Participants were asked to consider their mood over the previous seven days in their responses.

### Heart rate and blood pressure

Participants rested in a seated position for five minutes after completing the profile of mood states questionnaire. Afterwards, SBP and DPB were measured twice by the same investigator at the same time of day and on the same day and averaged using a manual sphygmomanometer (American Diagnostic Corp., Hauppauge, NY) and stethoscope. HR was measured manually at the radial artery for 60 seconds.

### Anthropometrics and body composition

Height and body mass were measured using a wall-mount stadiometer and electronic scale, respectively (SECA, Birmingham, UK). Total fat mass (FM), FFM, and regional (android and gynoid mass) body composition was determined by dual energy x-ray absorptiometry (DXA; model DPX-IQ; GE Medical Systems; Madison, WI) with participants in the supine position as previously described [17]. Coefficient of variation for body composition analysis using DXA in our laboratory for FFM and FM was 1.9% and 1.5% respectively, based on three repeated measures of 10 physically active young men. The quality analysis for the densitometer was conducted on a daily basis using a standard aluminum spine block (phantom) provided by the manufacturer. Measurements of the phantom were within the manufacturer’s precision standard with a coefficient of variation <0.5%.

### Cardiovascular and metabolic biomarkers

Venous blood samples (10 ml) were obtained before and after six weeks of RT and supplementation with MIPS or Placebo. Blood samples were collected in the morning on both laboratory visits as close to the same time as possible into either no preservative (serum) or EDTA-coated (plasma) vacutainer tubes (Becton, Dickinson & Company, Franklin Lakes, NJ) and centrifuged (IEC CL3R Multispeed Centrifuge, Thermo Electron Corporation, Needham Heights, MA) for 15 minutes at 3500 rpm at 4°C. Plasma and serum were then separated and subsequently stored at −80°C in 300 μL aliquots until analyzed. Blood was analyzed for total cholesterol (TC), high-density lipoproteins (HDL), TC/HDL ratio, non-HDL (TC-HDL), low-density lipoproteins (LDL), triglycerides (TRG) and blood glucose (Cholestech LDX Analyzer; Cholestech Corp, Hayward, CA). Inter-assay coefficient of variation was 2.1%, 4.0%, 4.2%, 4.1%, 4.7%, and 2.3% for TC, HDL, non-HDL, LDL, TRG, and glucose, respectively.

Total nitrate/nitrite (NOx) and cortisol were determined in duplicate and triplicate, respectively, using commercially available ELISA kits (R&D Systems, Inc., Minneapolis, MN, USA). High-sensitivity C-reactive protein (hs-CRP) was determined in triplicate using ELISA (IBL International, Inc. Hamburg, Germany). The intra-assay coefficient of variation for NOx, cortisol, and hs-CRP was 3.3%, 11.3%, and 7.4%, respectively.

### Nutrition analysis

Dietary analysis was measured using three-day food (two weekdays and one weekend day) records before training and during the last week of the study. Participants were asked to maintain their normal eating patterns and habits. FoodWise™ dietary analysis software (McGraw-Hill, New York, NY) was used for analysis. Participants were asked to mimic their pre-baseline day of eating prior to post-testing to the best of their ability based off of their dietary food logs.

### Reported side effects from supplements

Participants were asked at each exercise session about any supplement side effects. In addition, they were asked to fill out a questionnaire regarding any side effects at the end of testing (six weeks).

### Supplement analysis

To verify the absence of anabolic steroids, other illicit substances, and concentration of caffeine, the supplements and Placebo were sent to a third party laboratory for analysis (West Chester University of Pennsylvania, West Chester, PA). The samples submitted for analysis were mixed thoroughly and test samples weighed out. Each of these test samples was treated independently and studied in replicate. The analytes of interest were extracted into methanol using liquid-liquid extraction and quantitatively analyzed using gas chromatography–mass spectrometry (GC-MS). A Varian CP 3800 gas chromatograph- Saturn 2000 mass spectrometer with a Rxi-5Sil MS (30 m × 0.25 mm ID × 0.25 μm) column was used for the analyses. Standards of caffeine purchased from Cerilliant (Round Rock, TX) were used to generate instrument mass-response graphs used for quantitation.

### Performance outcomes

Performance data for this study have been reported previously [18]. Briefly, 1 repetition maximum strength (1RM) of the chest and legs was determined according to National Strength and Conditioning Association (NSCA) 1RM guidelines [19], maximal power was determined with a Wingate test (Monark Ergomedic 874-E, Vansbro, Sweden), and maximal isokinetic and isometric strength was determined with a Biodex System 3 Dynamometer (Shirley, New York).

### Resistance training protocol

All participants completed RT three-days per week for six weeks. The RT protocol was designed to target all of the major muscle groups (chest, back, trapezius, biceps, triceps, shoulders, legs, and abdominals) and was modified from our previous research [4,20] and that of others [13]. This protocol was chosen to allow for periodized progression in weight lifted while decreasing repetitions in order to continually challenge these well-trained athletes. In addition, we opted to follow a three-day protocol rather than a four-day protocol in order to provide the proper supervision of all RT sessions while allowing adequate recovery for the participants. Prior to each exercise session, participants performed a standardized five-minute warm-up on a treadmill. For weeks one and two, three sets of 10 repetitions of each exercise were completed at 70-75% 1RM. For weeks three and four, three sets of six repetitions of each exercise were completed at 80-85% 1RM. For weeks five and six, three sets of four repetitions of each exercise were completed at 85-90% 1RM (Table 1). Day one exercises included flat bench press, incline bench press, chest fly, latissimus pull down, seated row, and shrugs. Day two exercises included leg press, step-ups, leg curl, heel raise, lunge, abdominal crunch, and plank (held 60 seconds). Day three exercises included biceps curl, alternate curl, overhead triceps extension, triceps press down, shoulder press, and reverse fly. Rest intervals were 60–90 seconds between sets and 120 seconds between exercises. If a participant was unable to perform the prescribed weight for an exercise, the weight was adjusted to yield failure at or near the specified number of repetitions. The emphasis placed on consistent lifting form in this study, coupled with researcher supervision from certified personal trainers through the NSCA, helped ensure full participant compliance with training as well as reduce variability due to inter-subject differences or deficiencies in form.

**Table 1:** Resistance training protocol

**Weeks**	**RT variables**	**#**
1-2	*Sets*	3
	*Reps*	10
	*Load* (%*1RM*)	70-75
3-4	*Sets*	3
	*Reps*	6
	*Load* (%*1RM*)	80-85
5-6	*Sets*	3
	*Reps*	4
	*Load* (%*1RM*)	85-90

RT, resistance training. RM, repetition maximum. #, number.

### Statistical analysis

Data were analyzed using a one-way analysis of variance (ANOVA) to determine if there were differences between groups in baseline data. A 2 × 2 (group × time) ANOVA with repeated measures was used to determine the effects of the supplement and Placebo on the dependent measures. A Tukey post hoc analysis was used to identify significant differences when a significant *F*-ratio was obtained. Significance is reported at p < 0.05, and all values are reported as means ± standard deviation (SD). JMP Pro 9 statistical software (SAS Institute Inc., Cary, NC) was used for all analyses.

## Results

### Participant characteristics

Twenty-four participants completed the study. MIPS (n = 13) had an average age of 23.6 ± 3.5 years, height of 180.6 ± 6.7 cm, and body mass of 83.4 ± 11.5 kg. Placebo (n = 11) had an average age of 23.6 ± 4.6 years, height of 181.0 ± 4.7 cm, and body mass of 82.2 ± 7.2 kg. Groups were not different at baseline (Table 2). Participants were monitored by research personnel with NSCA certifications for all training and supplement consumption on training days. As a result, 100% compliance was observed for these days with respect to both RT and supplement consumption. On non-training days, participants verbally reported 100% compliance for supplement ingestion and returned all empty single-serving supplement containers as evidence. As previously reported [18], our periodized RT protocol resulted in significant improvements in 1RM strength in both MIPS and Placebo participants indicating that the protocol was effective in increasing strength.

**Table 2:** Participant characteristics at baseline (n = 24)

	**MIPS**	**Placebo**	**p**
n	13	11	
Age (years)	23.6 ± 3.5	23.6 ± 4.6	0.958
Training (years)	6.1 ± 3.4	4.5 ± 3.7	0.291
Height (cm)	180.6 ± 6.7	181.0 ± 4.7	0.856
Body Mass (kg)	83.4 ± 11.5	82.2 ± 7.2	0.767
BMI (kg/m^2^)	25.4 ± 2.2	25.1 ± 2.0	0.664

Data are mean ± SD. MIPS, multi-ingredient performance supplement. Placebo, carbohydrate placebo. BMI, body mass index.

### Profile of mood states

No group × time interactions were observed for any profile of mood state variables (p > 0.05). However, there was a main effect of time (p = 0.014) by which training resulted in an increase in vigor regardless of group (MIPS: pre 15.6 ± 5.8, post 18.1 ± 6.9, +16% vs. Placebo: pre 17.3 ± 6.9, post 20.1 ± 4.7, +16%).

### Blood pressure and heart rate

SBP (MIPS: pre 120 ± 10 mmHg, post 117 ± 14 mmHg; Placebo: pre 116 ± 7 mmHg, post 115 ± 10 mmHg) and DBP (MIPS: pre 74 ± 10 mmHg, post 72 ± 10 mmHg; Placebo: pre 69 ± 8, post 67 ± 9 mmHg) were unchanged as a result of training in both groups. Resting HR also did not change as a result of training or supplementation (MIPS: pre 62 ± 8 bpm, post 66 ± 12 bpm; Placebo: pre 59 ± 6 bpm, post 61 ± 11 bpm).

### Body composition

**Figure 1:**
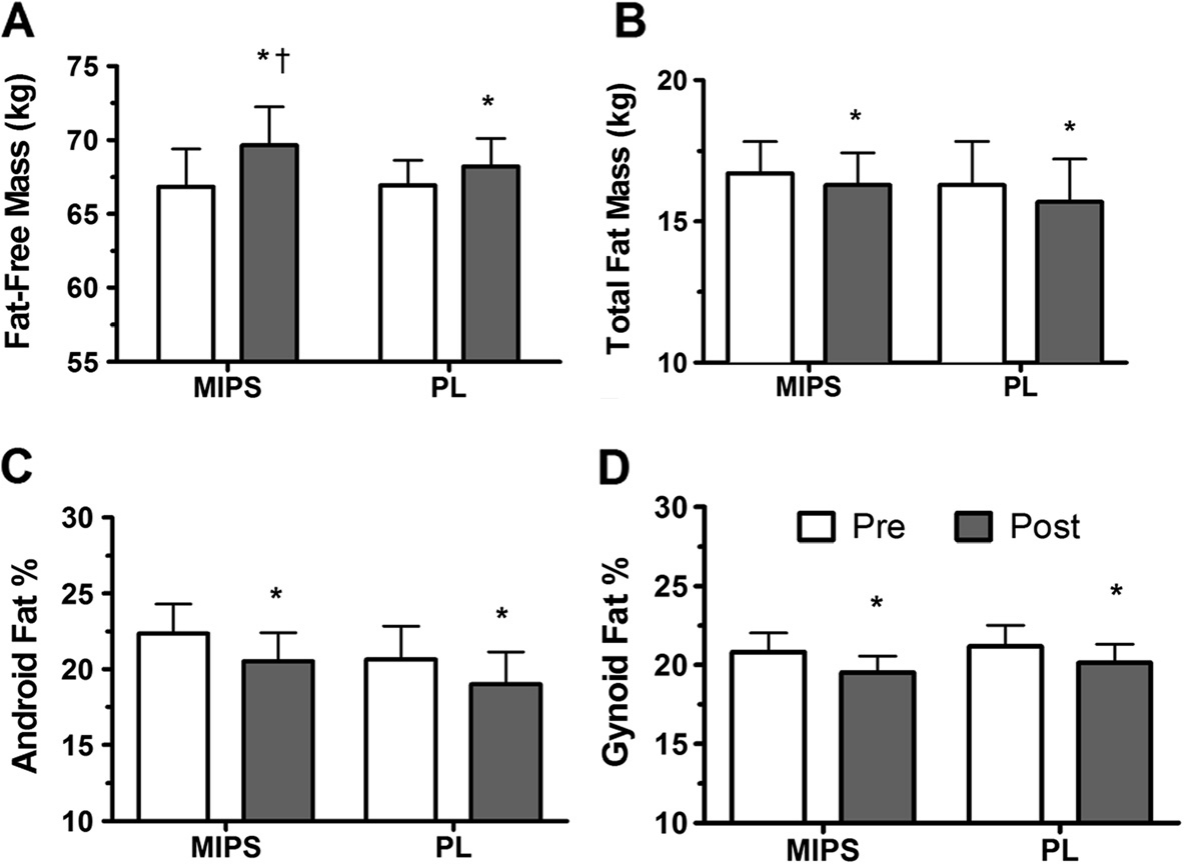
**Body composition pre and post six weeks of supplementation and resistance training. A**) fat-free mass, **B**) total fat mass **C**) android fat percent, and **D**) gynoid fat percent before and after six weeks of resistance training and supplementation with a multi-ingredient performance supplement (MIPS) or a carbohydrate Placebo pre- and post-exercise in resistance-trained men. White bars, pre-testing; Dark bars, post-testing. *, P < 0.05 compared to baseline. †, P < 0.05 compared to Placebo at the same time point.

Total body mass (Table 3) was increased in both groups (p < 0.0001) but more so in MIPS than Placebo (p = 0.024). FFM (Table 3) was increased in both groups (p < 0.0001); however, the increases were significantly greater with MIPS (p = 0.025). Total body (p = 0.0005), android (p = 0.0009), and gynoid (p = 0.002) fat percentages all significantly decreased over time, with no group by time differences (Figure 1).

**Table 3:** Body composition at baseline and after six weeks of supplementation and resistance training (n = 24)

**Variable**	**Group**	**Baseline**	**Post**	**Time**	**Group x Time**
Body Mass (kg)	MIPS	83.4 ± 11.5	85.8 ± 11.8	<.0001*	**0**.**024**^†^
	Placebo	82.2 ± 7.2	83.3 ± 7.7		
Fat Free Mass (kg)	MIPS	66.8 ± 9.2	69.6 ± 9.2	<.0001*	**0**.**025**^†^
	Placebo	66.9 ± 5.3	68.2 ± 6.0		
Body Fat %	MIPS	20.7 ± 4.0	19.5 ± 3.8	**0**.0005*	0.682
	Placebo	20.2 ± 5.0	19.2 ± 4.8		
Android Region Fat %	MIPS	22.3 ± 7.0	20.5 ± 6.8	**0**.0009*	0.846
	Placebo	20.6 ± 8.7	19.0 ± 8.7		
Gynoid Region Fat %	MIPS	20.8 ± 4.3	19.5 ± 3.7	**0**.0019*	0.666
	Placebo	21.1 ± 6.1	20.1 ± 5.9		

Data are mean ± SD. *Indicates a significant main effect of time. † Indicates a significant difference between groups (p < 0.05). MIPS, multi-ingredient performance supplement (N = 13). Placebo, carbohydrate placebo (N = 11).

### Cardiovascular and metabolic biomarkers

Blood markers of CV and metabolic health (blood lipid profile, glucose, cortisol, NOx, and hs-CRP) were within normal clinical ranges for the entirety of this study. There were no time or group × time (p > 0.05) effects observed (Table 4) for any of these measures. The sample sizes differed slightly between biomarkers because some participants’ samples were not useable or blood was unable to be drawn.

**Table 4:** Serum measurements

**Variable**	**Group**	**n**	**Baseline**	**Post**	**Time**	**Group x Time**
Triglycerides	MIPS	10	90.6 ± 27.1	118.5 ± 41.6	0.1124	0.571
mg/dL	Placebo	7	89.3 ± 26.9	102.9 ± 47.8		
Total Cholesterol	MIPS	13	150.4 ± 31.4	145.0 ± 18.6	0.9464	0.273
mg/dL	Placebo	9	162.8 ± 47.5	167.6 ± 41.5		
HDL	MIPS	13	44.5 ± 7.4	42.6 ± 8.2	0.3284	0.978
mg/dL	Placebo	11	45.0 ± 13.2	43.1 ± 6.6		
LDL	MIPS	10	89.5 ± 29.0	82.7 ± 19.4	0.9253	0.201
mg/dL	Placebo	7	103.7 ± 29.7	109.5 ± 46.6		
Non-HDL	MIPS	13	105.9 ± 30.0	101.2 ± 21.1	0.9725	0.249
mg/dL	Placebo	9	117.8 ± 47.6	122.2 ± 43.5		
Glucose	MIPS	12	94.4 ± 4.0	97.1 ± 10.1	0.2655	0.821
mg/dL	Placebo	10	92.1 ± 10.4	94.0 ± 6.8		
Cortisol	MIPS	13	123.4 ± 57.2	123.4 ± 65.8	0.5071	0.503
nmol/L	Placebo	11	116.2 ± 37.1	98.4 ± 31.5		
Endogenous Nitrite	MIPS	13	5.3 ± 1.6	5.2 ± 1.1	0.9663	0.761
(μmol/L)	Placebo	11	5.3 ± 1.2	5.4 ± 1.4		
Total Nitrite	MIPS	13	18.4 ± 18.5	17.9 ± 18.0	0.7688	0.957
(μmol/L)	Placebo	11	20.6 ± 20.6	20.2 ± 20.2		
Nitrate	MIPS	13	13.1 ± 6.5	12.7 ± 11.0	0.7782	0.978
(μmol/L)	Placebo	11	15.3 ± 6.5	14.8 ± 9.1		
Nitrate/Nitrite	MIPS	13	0.5 ± 0.4	0.7 ± 0.7	0.3325	0.629
	Placebo	11	0.4 ± 0.2	0.4 ± 0.3		
hs-CRP	MIPS	11	1.0 ± 0.87	1.1 ± 0.97	0.1478	0.199
(mg/L)	Placebo	8	0.78 ± 0.89	2.1 ± 2.7		

Data are mean ± SD. HDL, high-density lipoprotein; LDL, low-density lipoprotein; Non-HDL, total cholesterol minus HDL; hs-CRP, high-sensitivity C-reactive protein. MIPS, multi-ingredient performance supplement. Placebo, carbohydrate placebo. Sample size between each analyte is slightly different due technical difficulties with certain samples.

### Nutritional analysis

Data regarding nutritional intake have been reported previously [18]. Briefly, all participants were asked to complete a three-day food record before RT and during the final week of RT, only eight of the participants (MIPS, n = 5; Placebo, n = 3) returned satisfactory food records to the research staff for analysis. However, the participant population included only highly resistance-trained individuals and thus, they verbally reported monotonous eating patterns and were instructed to replicate pre-baseline testing nutritional intake for post-testing. Briefly, total Calories (MIPS, 37.6 ± 8.3 kcal/day/kg vs. Placebo, 25.3 ± 5.8 kcal/day/kg, p = 0.34), carbohydrate (MIPS, 3.2 ± 0.7 g/day/kg vs. Placebo, 2.5 ± 0.8 g/day/kg, p = 0.49), protein (MIPS, 1.9 ±0.5 g/day/kg vs. Placebo, 1.4 ± 0.3 g/day/kg, p = 0.56), and fat intake (MIPS, 1.8 ± 0.8 g/day/kg vs. Placebo, 1.0 ± 0.9 g/day/kg, p = 0.51) for this subgroup (n = 8) was not different at baseline, remained unchanged for the duration of the study and was not different between groups [18].

### Performance data

Performance data have been reported previously [18]. Briefly, no differences existed between groups for training volume and both groups had significant increases in 1RM strength for upper and lower body with no difference between groups (Leg press, MIPS: pre 336 ± 24 kg, post 418 ± 25 kg, p < 0.001; Placebo: pre 318 ± 28 kg, post 429 ± 29 kg, p < 0.001; Chest press, MIPS: pre 112 ± 7 kg, post 123 ± 7 kg, p =0.001; Placebo, pre 118 ± 8 kg, post 127 ± 8 kg, p = 0.001).

### Reported side effects

Over the six-week study nausea and dry-mouth was reported by one participant in MIPS. Feelings of paresthesia were reported by two participants in MIPS and two participants in the Placebo group. No other side effects were reported.

### Supplement analysis

Caffeine was only present in the pre-workout supplement (SHOT; 190 mg ± 10 mg per 21 g serving). No additional stimulants were detected in either supplement. No quantifiable amounts of caffeine were detected in the post exercise supplement. These data are in agreement with the supplement fact sheet listed by the manufacturer. No other adulterants such as commonly abused stimulants and known anabolic steroids were detected.

## Discussion

The primary findings from this investigation were that six weeks of MIPS during RT did not alter CV or metabolic health (blood lipid profile, SBP, DBP, HR, glucose, cortisol, NOx, or hs-CRP) in resistance-trained men. Feelings of vigor and maximal strength increased in both groups regardless of MIPS or Placebo. In addition, six weeks of MIPS in resistance-trained men appear to improve body composition to a greater degree than Placebo. This is in agreement with the results of others who have reported similar findings in untrained participants [12,13]. These studies also reported an improvement in muscle mass and body composition in untrained men without any deleterious effect on blood safety markers when consuming MIPS either before or before and after RT for four weeks [12,13]. It has not been determined until now, however, whether MIPS impact CV (e.g. blood lipids, BP, and HR) and/or metabolic (e.g. cortisol) health and body composition in resistance-trained men (a primary marketing target of this class of ergogenic aid). For this reason, we sought to investigate the effects of six weeks of pre- and post-RT MIPS consumption with periodized RT on CV and metabolic health and body fat in resistance-trained men.

Changes in body composition in the current study were dramatic and in some variables, greater than expected based on the current literature. We observed an increase in total body mass in both MIPS (2.9%) and Placebo (1.3%) with MIPS exhibiting a significantly greater gain than Placebo (p = 0.02). Participants in MIPS also increased FFM significantly more than Placebo (+4.2% vs. +1.9%, respectively, p = 0.025). Similar to Shelmadine et al. [12], these gains mirror increases in FFM measured in untrained men after four weeks of RT with a pre-workout only performance supplement that was the same as was used in the present study (SHOT, +4.8% vs. placebo, + 1.7%). In addition, Spillane et al. [13] reported similar findings of increased FFM in untrained men after four weeks of RT when supplementing both pre- and post-RT with the identical performance supplements used in the present study (+3.7%) compared to placebo (+1.0%). It is surprising that our findings so closely mimic the results reported in untrained populations despite our participants averaging 4–7 years of RT experience and substantially higher baseline FFM (+9-13 kg) than participants in the aforementioned studies [12,13]. The increase in FFM is most likely due to the creatine and/or β-alanine in the product; however, due to the proprietary nature of this supplement, the exact amounts of these ingredients are unknown in the product.

Body fat percent decreased significantly in both groups and this was not solely a function of increased FFM. Total body fat decreased by 2.6 and 3.5% in MIPS and Placebo, respectively. In addition, android (MIPS: -1.8; Placebo: -1.4%) and gynoid fat % (MIPS: -1.3; Placebo: -1.0%) decreased significantly in both groups. Android fat is highly associated with increased risk of CV mortality and diabetes mellitus and thus, it is notable that our RT protocol was successful in reducing both android and gynoid fat. Our findings are supported by research demonstrating that exercise can decrease body fat independent of supplementation [17,21]. Our prior work has shown increased lipolysis with just one bout of RT in resistance-trained men [4] and in overweight/obese sedentary men [20]. A gain in FFM with concurrent decreases in body fat is a highly desirable goal of athletes in many different sports as well as recreational weight-lifters. Our findings support the supplementation of MIPS in combination with periodized RT as an effective method of improving body composition.

Regarding the multitude of individual ingredients in the proprietary blend of SHOT and SYN, including whey protein, BCAA’s, creatine, caffeine, β-alanine, and L-arginine, it is difficult to isolate their specific effects. In fact, some research suggests a synergistic effect of ingredients in the investigated supplements [22-24]. The increases in FFM we observed may likely be explained by studies that used supplementation of creatine with β-alanine [22] or whey protein and amino acids [23]. Hoffman et al. [22] demonstrated that RT in combination with supplementation of creatine and β-alanine decreased body fat by 1.2% through an increase in lean body mass (LBM) (1.74 ± 1.72 kg) greater than creatine alone (data not reported) or a carbohydrate placebo (0.44 ± 1.62 kg) in collegiate football players. Willoughby et al. [23] observed an increase in FFM of 5.6% when combining 14 g of whey and casein protein with 6 g of free amino acids as compared to a 2.7% increase with a carbohydrate placebo supplement in untrained men following 10 weeks of heavy RT (3 sets of 6–8 reps with 85-90% 1RM, 4×/wk). Perhaps most relevant to the present study, Schmitz et al. [24] recorded 2.4% and 0.27% (p = 0.049) increases in LBM in trained participants (≥ two years RT experience) supplementing with MIPS with and without BCAA’s, respectively. The investigators implemented a nine-week progressive overload RT regimen with participants training four times a week. The two supplements were matched for creatine (4 g), protein (7 g), and carbohydrate (39 g). The supplements from the present study include the primary ingredients from these studies [22-24] and alternative ingredients in a proprietary blend that are purported to improve body composition. Our results suggest that the ingredients in SHOT and SYN may be working together to improve body composition greater than the Placebo.

All measured CV and metabolic variables were within normal clinical ranges at baseline and post-six weeks in the present study. Interventions using various intensities of RT with untrained participants have significantly lowered LDL cholesterol in the same six-week span as the present study [25]. While RT may also improve TRG in untrained individuals [26], our participants were experienced with RT, which likely contributed to the lack of difference measured in these variables. Our findings are supported by previous studies in untrained men [12,13] using SHOT and SYN, which also report no changes in CV health variables after 4 weeks of RT in men of similar age to participants in the current study.

Cortisol is widely accepted as both a catabolic biomarker and an indicator of overall stress. Based on the current literature, we hypothesized a decrease in cortisol concentration in MIPS but measured no changes. Bird et al. [27] reported that both carbohydrate (6%) and carbohydrate (6%) with essential amino acid supplementation (6 g) during an acute bout of RT suppressed the cortisol response in untrained men compared to a placebo. This may have been due to a glucoregulatory effect in which carbohydrate was an adequate source of energy to prevent the need for cortisol to stimulate the liver to release glucose. Sharp and Pearson [28] demonstrated decreased cortisol concentrations in recreationally active young men after three weeks of BCAA supplementation (6 g/day) followed by a fourth week of supplementation and four bouts of RT (three sets of 6–8 repetitions with 80% 1RM and 60 seconds between sets and exercises, three lower- and five- upper body exercises). The authors reported significant decreases in cortisol 12 hrs after RT bout two, and 12 and 36 hrs after RT bout four. Because of the low-training status of the participants, the RT was deemed an over-reaching training week. Two factors that may have contributed to the differences observed in our data are the experienced training status of our participants and the slightly later (36 vs. 48 hrs) post-training cortisol measurements in the present study. Our data indicate training-status may be a determining factor in resting cortisol concentrations with performance nutrition supplementation.

Total nitrate/nitrite (NOx), which is indicative of production of the potent vasodilator, nitric oxide, was also measured to evaluate the impact on CV health. Nitric oxide is synthesized from L-arginine and L-citrulline, two amino acids present in the MIPS in the present study. Nitric oxide has been reported to improve CV health through vasodilation and prevention of lipid build-up on the arterial walls [29]; however, this finding is specific to diseased populations [30]. We recorded no change in resting NOx within or between groups during the intervention, which agrees with most previously published research [13] in healthy individuals. Interestingly, L-arginine has recently been shown to increase blood volume within the muscle (measured with near-infrared spectroscopy) without changing NOx [31]. It is possible that our participants experienced similar changes in blood volume without the detection of any change in NOx. In addition, by design, we sampled blood 48 hrs following the last training bout to measure the impact of supplementation and training over time rather than acute response to RT.

The inflammatory marker hs-CRP was measured to reflect total body inflammation. Clinical data indicate values of <1, 1–3, >3 mg/L as low, moderate, and high risk, respectively, for CV disease. We recorded no significant changes in hs-CRP within or between groups. Kadaglou et al. [32] recently reported no change in CRP following three months of RT (3×/week) in type two diabetic patients. Conversely, Sheikholeslami et al. [25] demonstrated significantly lower CRP values in untrained men following six weeks of moderate (45-55% 1RM) or high intensity (80-90% 1RM) RT in healthy males compared to a non-intervention control group. The discrepancy in results may be due to the experienced training status of our participants compared to diabetic and untrained individuals. In addition, CRP is notorious for its high intra-subject variability [33] and may have confounded our results.

While RT may independently improve BP in untrained normotensive and pre-hypertensive individuals [26], our data did not indicate changes in BP, perhaps due to our participants’ RT history. Similarly, we saw no changes in HR, likely due to their training status. Research indicates that caffeine, a central ingredient in SHOT, the pre-exercise MIPS used in the present study, can increase HR, SBP, and DBP acutely [9]. Energy drinks with less caffeine than SHOT (80 vs ~190 mg, respectively) have been reported to elevate BP up to 24 hrs after consumption in nine healthy (aged 18–45 years), nonsmoking, normotensive men (n = 4) and women (n = 5) who self-reported to consume between four and 379 mg/day of caffeine (four participants reported habitual caffeine intake) [34]. However, some research suggests chronic coffee drinkers are at a decreased risk for developing CV disease [35]. No published research indicates the HR and BP response to caffeine-containing MIPS.

Regarding mood states, RT elicited an increase in vigor for both MIPS and Placebo. This indicates that it was unlikely our participants were overtrained at the post-testing time point. Bresciani et al. [36] reported a non-significant decrease in vigor and a significant decrease in total mood as being indicative of overtraining in active men.

Resistance-trained men appear to tolerate SHOT and SYN better than their untrained counterparts. Indeed, Shelmadine et al. [12] reported feelings of dizziness, nausea, headache, rapid HR, shortness of breath, and nervousness after consuming SHOT in untrained men. Similarly, Spillane et al. [13] reported select untrained participants experiencing the same side-effects while supplementing with both SHOT and SYN. Only one individual taking SHOT and SYN in the present study reported feeling nauseous while two others reported feelings of paresthesia (although two people in Placebo also reported feeling paresthesia). It is probable that individuals experienced in RT are also experienced with a variety of MIPS and may be more tolerant or less likely to report side effects if any were present.

### Limitations

The present study was limited by the accuracy of self-reported dietary intake and supplement consumption on non-training days. However, our research staff collected empty supplement single-serving Ziploc® bags three times per week in an effort to verify compliance and all participants verbally reported no change in eating patterns. Due to the small sample of useable nutrition logs, it is possible that we did not have a representative sample to determine no differences between groups. However, all participants were questioned personally and reported no change in dietary habits for the duration of the six-week study. In addition, differences in prior training status of our participants may have limited our findings; however, even though MIPS had a longer training history (Table 2), they still were able to increase FFM more than Placebo. In addition, those in the MIPS group may have been able to recognize the feelings from the pre-workout supplement that contained caffeine and β-alanine and known they were in the supplement group. Moreover, participants were not monitored 24 hours per day and thus may have been able to discuss how the supplement felt to them. However, all training was completed with limited contact to other participants and any interaction would have been outside of workout and/or laboratory visits. Lastly, we do not have reliability data for the POMS assessment used in this study.

## Conclusions

Considering the popularity of MIPS, it is important to confirm their safety and efficacy. Six weeks of MIPS before and after RT does not alter HR, SBP, DBP, fasted blood lipids, glucose, cortisol, NOx, or hs-CRP in healthy resistance-trained men. However, FFM was increased to a greater extent in MIPS than Placebo and improvements occurred in regional body fat and feelings of vigor, regardless of treatment group. Future studies investigating the effects of MIPS on cardiometabolic health variables and body composition should consider closely controlling diet, extending supplementation beyond six weeks, and including female participants and individuals older than 40 years of age.

## Competing interests

This study was supported by an independent research grant and product donation from VPX Pharmaceuticals (Davie, FL). None of the authors had financial or other interests concerning the outcomes of the investigation. The authors declare that they have no competing interests.

## Authors’ contributions

MJO conceived and designed the study, secured funding for the project, provided oversight of data collection, analysis, biochemical assays, and manuscript preparation. DDT carried out all subject recruitment, data collection, exercise training, immunoassays, and assisted with manuscript preparation. WKM carried out data collection, participant recruitment, and exercise training. AWK, EGW, SH, and ES helped with data collection and analysis. LBP, TPS, and JSK provided assay support, and insight into drafting the study design and manuscript. All authors read and approved the final manuscript.
